# Supplementary material for: Four Novel Mycoviruses from the Hypovirulent Botrytis cinerea SZ-2-3y Isolate from Paris polyphylla: Molecular Characterisation and Mitoviral Sequence Transboundary Entry into Plants
Source: Viruses. 2022 Jan 14;14(1):151. doi: 10.3390/v14010151 (PMC8777694; doi:10.3390/v14010151)
Supplement: Supplementary file 1 [file viruses-14-00151-s001.zip › viruses-1505710-SI/Supplemantary File S1.pdf]

Supplementary File S1. The sequences information of six viral contigs from *Botrytis cinerea* SZ-2-3y strain used in this study.

>Contig 111 (2992 nt)

CTTACGAAAACGATGAATCACGGGCGGGGGGTCCTGACCATTTCTGATCAATAAATCCC
GAGCTTTCGCTCTTTATTTATTTTAAGGATGATGTCCAGGACGTGATCTTGGTATGATCA
CCCAAGCGCATATACCGTTAGAACGAACCACCTGACTGTGTAGGGGAATTAACTGAAT
AACTACCCTGTTTTATAACAGTGGATAAGATGAAATACATTCTACTTACAGTACGAACTT
TCAGCTGGCAAGACTAATCATGAAGGCTTGGCCCCTTACCACCGGATCTTGATTAAACT
CACTTCTTTAGAAGGAAGTCTGTCAAGACGGCAATGAGAGACCTATACCTATAGCCCAT
CAAGTGATGGAATACTAAATCTTTAGACTTCTATGAAAACATCTACTTTAACAATTTTAA
ATTCTTTTAAATTATTAAAAAGACGTTCTTTAAGAAGTTTCAAGATGGTATCTCTCGCCA
AAATTGGCACGATCAAAAGCGTGTTCCCAAAGTTAATTTCATTATCTTTGGGCCGCTTA
GATCGTATTAAAGCAAGAGTCCGGATGTCTAACAACTTGATACAGTTCATCCAGCGTAT
GTCTGTTCACCACGGTGACGCTTATACCATAAAATGGTTAAAGTGTTCTTCCGTAGCTTT
ACAGAAATACATTGCTGGTGATCCGATTTCAAGTTTAAGATCTCTTGAACCTAAAATTC
CTCTTCCACGGTTAATAAACGGTTGTCCTAGTATAATTTCTAAGACAGATCGTAAGTTAA
TACGTGAAGGGAATACATGGGTGAGACGTTTTTGATTGACACAATTCGCTCTTTATAGG
GTTTTAAAATTCCCTAGTAGAGTTGACTTGTCATCGATCACAGATCCTTACTCAGGTTCA
AGTTCCTTTCTGATACAGCAAATTCAGTTGGCTAAGGATTTTAATCCTTTTGCAATATAT
GCAAAGGATTTCAAATTCCTTCCTCCAACTACCCTTGTTTTCTCTCAGAAGTCTTCTCC
CTCCAATGGGATTTCCTATCAAGGAATTCTTACGGATTATCATAACCTCTCTCGAGGTTC
ATGAATTTTAGGAGAGGACATTCTCTTTTAGAGAATCTTAAGATTAATGGTATGAAATTA
CCTTCAAAGTCAAGTATCTTTGGAACTGGTTTATCTCAGTTCGCGTTGAAACACGAGA
GTGCTGGAAAGATTAGAATCTTCGCATTGCTGGATTCTATATCTCAATCAGTACTTCGTC
CTCTTCATGATTCCTTATTTGATATACTTAGATGTATTCCAAATGACGGAACTTTTGATCA
GGACGCCTCGGTTTCCCGGTCTGCGGATAAACTGGCTAAGTATGGAGTGGCATATTCTT
TGGATCTTTCTTCTGCAACAGATAGGTTACCTTCAAGGCTAACAGCCCAGATCCTACAG
TCGATTTTATCGATTGAGGGATTTGGTGATGCTTGGCTTAAGGTAATGATCGATAGAGAC
TTTTGTTTATCGTCTCTTGATCAAGTCTATGCATTAGAAGGTGAAAAGTATATACCCCAA
TATTTTAGATATTCTGTGGGTCAGCCCATGGGTGGTCTAAGTTCGTGAGCCGGATTAGCT
ATTACTCACCATTGGATCATGCAAGCGGTCTCTCTTCGGTGCTCAAGAAAGTTTTCTAA
TCTTTCTTTGGTCACTTGAGAGGATCGTTACGAGGTCCTTGGTGATGATATAGTTATCTT
TGATGAAAATCTTGCTCAAGAGTACCTAATATTTATGAGAGAACTTGGTGTCGGTATAA
ACCTAACTAAGTCTCTTTCTAAATCATCAGATACTTTTGAGTTTGCTAAAAGAACGATTA
GTAGAGGTGTTAACATCTCTGGTTTATCGTTCCAACAAGCGCTTAGTTCTTCATCTTTAG
GATCACGAGTTTCAGATGCTTATACTTACAGCTCTTTAGGGCTGGTAAGGACAGCATCT
CATCTTGGACATCTATTGGCGGCAAAACCGACTTCATCTTCTTTTAGGAAGATGAAAGA
GATCGGTTTGCCAGCTCTGTCACTTTACAATCTTTTATTCTCAAAAGAGATAATAGAGTT
GGAAAGAGTACTAGAATGTATTGTCAATCCTCGATTTGAGGATTTCGACTTTGAGAAGG
CCAAATTTGACCTTCCTTTGCATTCTATGCTACGACATTGCTTAGATCTAATACGTCTAA
AAGGGTCTAACACAAGTGAGTGAGGATTGGCTCCTTCCGGAGTTCTTCCTTCTTCCTTG
GCGTACCCCTTTTCTCGTCAGGATGACCGAGAAGAAGTCTCTTCTGAGTACGAACCGC
ATTTAGTTGCAGTTATACTTCAGGAGGCTCTAGCTAAGTCTAAAACCTTAGTTAGAGATT

ACGAGAACTTGATCAAGAGAGGTGCAGTTACTGTCTATAAAGGACAGGGAACTAAACT
TCTACAAGCTCAAGTCTCGGGTTTCTTCGAGGATCTAATTATTGAGTTTTCTGATTTAGA
TGTATCGGACTTCTGTGATGAAATAGAATCAATGCTATATCGTCACGCGAAGTATCCTAA
ATACTCTATATCAGAAGCTCTCTCTACCTTAGATCGGGTAGAGAATCTGATATTCCGTTT
CACTTTTAAGACAGAGATGTCTCGTTCAAAATATGAACAAGATTCTTCTCCGATCATAA
AGATGTTGCGGAAATCAGAGGGTAGCATTCCCATTCCATACTGACAGACATCCGCTCTT
CCTTAAATGATGATATATCATCCTTCTTAAAGGCGTTGACGAATTTCGTTAACGTCTCTA
ATTTAAAAGAGGGTCCCCAAGATTACTGTAAAAAGTAGGCAGGGGGAGTGCCCTTCAT
ATTTCATGAGAATGCCCCCACCTTACCCCTCGTCGCGGCTTTGTGCCGACAAAAGCTAA
TTAATAAACAACAATCTTTCGTCGCTCACGACACTTCATCACTTTGACGTCTATAATCAA
CATGTCTGGGTATCCGGGAT

>Contig 334 (2777 nt)
TTCCCTTCGTTTGTAAAACATAAGGCTCAACGTGCATTCCCGATACCGACGATAGGATG
AAACCTTCAGACGATTCTATTGGGAATTCAGGGAATACCAGAAATGGAGGTACCTGTA
CCCGCTGCCCAGCAGCTGGGTCCCTGTCAATTGCTCTCACACGAGCAGTTCGTATCATA
CGATTGGAATATAAGATTCCGAATGATATTTTACTTCCTAGGAAGCAGAACTGCACGAG
CCTAAGAGCTTCATGGAATGAGTGGACGACTTCAGCACAAAGCTGTGTGAATGTCAAT
AAGCCTAGAGGTAGAAGGCTAGCTATGTTGGTTAAGTCCGGCAACCGGCTATTCGATCT
GCCTTGCAGAAGCTGTGACAAGTCACTAAGCTGGCAAGCAGAACAGGACTGGATAAG
GAGCGTGGGTAAGCCAACTCCTAACGATCTTGTACCAACGATCTTTGATCTCTTTAAAC
TTAGAGAGAAGGTCAGACAAGAAGTCAGAGGATGGGGAAGGAGACTCGCATCGGCG
AGAGACAGTTTGGAGGAACCGTGTTTGGGCGACTACGTCCCGGGGGTTCAAGGGTGT
GCAGAATTGAACACAATGAGTGGCGGAACCTTAGGAGTCTCAGAGAGTGAATATAGCG
GCCAGCGCAATGTGGTCAGACTGGGATGTGCAAAAACAAAGGGAAAGTTTCGTGTTG
TGACAATGCAATCTGCGGAGGTCAAGAGAGTCTTGACTCCACTGCATAATGCCTTGTAT
GGACACATCTCATCGAAGGGATGGTGTGTCCGGGGGGACGTTTTAAGGGGGGACTTCG
AGGAGATTATCGAAGATCGAAAGGAAGGTGAGAAGTATATTAGTGGCGACTATAAGAG
TGCTACTAACAAAATTTATACAGAAGCTGTACTAACGATAGTCGATGAGATCTCCAGGA
CCCCTGAGCTCACAGAATTGGAACGAGAAGTGTTTGTGGAATCCTTTACGGATATGTG
GTGGTTCAAGAGACTTGAGAGCGGACCTATGTGTAGGGGCTCTCCCATGGGAAGTTTG
GTAAACTTTCCGATGCTGTGTATCTTGTTTTGAAATCGCCTGCGATAACGTAATAGGCTT
GCGCCGCAAAGAAGTAAAGAGGGTGAAGATTAACGGAGACGACATAATGTTCGCCGG
CACCGCTGTTTTGTACCAGGAATGGCGACGTGTGACCGGAATTTATGGCCTTGAAGTTA
ATGAAAGTAAAACCGAGATCTCGGATAGGTGGTTGGACCTGAATAGTCAGTCCTTCGA
TACCTTTCGGAGAAAGATGGTCGCAAAAGCGACACTCGGATTTCTTCGCCCTAATCGA
CAAGAACCAGGCGCATTGTTGCGCGCCATCATTGTGGGTATGAAAGGTTTTAAGACCG
GTCATATTATGCAAGTGATCACTGTGCTCCGGCACGAAGTGGCTTTGCGTGGTGTAACT
GAGGATCTTACGGAAATCGGACCCTATTGGCGAAACGCTCTCGTGAAGAAGAAGTGGT
TTCGGCAAGCACTTGCAGTTGGCAAGTGCCCCGAAATCAGACGCGGTCAAGACCGGT
CGCTGGCAGTTGAGCCAGGGCCACCTCCACGTGAACGATTCTATGCAGTTATTACCCGC
ATCGCCGCGCAGGCGCAGAGTGATAACACAAAGGAGTGGACAGGCGTTAAGGTAAAG
AACCTTACTGTTCGCCTAGACAGAAAAGCATGGAAGAATTTGACAAAGGAAAAAGCT
GACCTCGCCGATAGGAGGTATATTTGGAAAGGAGTCCGTTGGAGTTTCTTGTGGCCCA

AGGAATTACTCACCCTAGTCAGGGAGTATTTTCCCCGGATACTGGAAACCAAGACGCC
AAAATGGATGACTGACCACCCTTTCTTAACGCGCTCGCCTGTAGGTCGCGTTGAGAAG
AAAAGAAAATATGCCTCATGCTCTCCCCCCTCGTGTCTCCAACCTGTGGAGTTTGTTAC
GAAGTGGGAAAGGGAACAGGCTGAATGGGCAAAGGAAATGTCGATGTCTGTGTAGAC
GGTGGTCTCACGAGGACGTCATGAAATTATGGGCCGAATGCTTGATTCGAACCGATTCA
AGAGGTGTCCACGAAAGGAGTCGCATAAGGATGGTAAATCGGATTGCCAGTACGGCTC
ACCGCAGCAGAGTAGGGAGACTCACTATATCATTATATATTGGCCTCAAAGAACTGAAA
TGACAGACGAAACTGGGAGAGTTTTAACGAAGGGAAGTGCTCTAATGGTTAACGGTA
GGATCGCTAGTGGGTATCCAGCTACTTAAGTGGATAGAAATCCGAAAGGACGTTAAAA
ATTGTCAACCGGTGACCCTCGTGAGAGGTGAGCTAACGGGGCGGATAAGTCCTCGTCG
ATCATTGTAGACCGCATTAGAAGTACCAGCGTAGTTAGATCTCGTCAATGGAAAACCAA
GCGCACGGGGTTTCAGGAAATGAGTGTAAAGTCAATCGCTTGATAGGGGTCGTTATGG
AAAACCTATATGCTGATTAACGGTGTTGTACTAAAGACTGGTAACGTATGCCTGCATAC
GAGTGCCGCACGGAGTGCGACGATTGGAGTGGTTCCTTAAGATGACCGTAAGGATGAC
GGTAGAGAGGAGTTAATGAATTGACTAGTGAGTCACCGATGCAATTTCATCGGATTTCA
CTGCAGATGTTGCAGACCAGCGGCAATGGATCCTGGAGTCAACGCGAAAGCGTGGGT
GCCCGCTACCCCCGGGGTAGACACCCCACTCAACTCCCGCCCACA

>Contig 420 (2802 nt)
GCACAATTGGTCAGAGAGGAGCTTGATTCCGCATTCCGATCGTGTCGAAGTTGCATTAT
GAGTAACCATTCGTCATTGATCAATGCCGCGCCTACCTGTCAGGGTGGAGCGTCTCTTG
TCAAGTCTCTTAAAGTTGTAGCTGAAACTGTGTCTCGTGAATTTGGATTCACCACAGGC
CCTTGTATAAAGGGTGCCAACTGCATAACCATAAGAGAAGAATGGGATGGATGGATCA
AGGATTGTCTTGCCAACCAAGTTTATTGGAGCCGAGTGCGACATGCACCCAGCAGAAA
AAGAAAGTTTGTTTCCCTGTCAAAGGGGGTCAAGCGAGTATTTGATGCTACTTGCAAA
CAGTGCGACAAAAAAGCCTCGAGAGAGGCAATCAATAAATGGACAAAGAAAATGGCT
GAGGATTGCCGTGATGGGGAAACCCATTGCTCCAGGTACCTTGATCAGTTGAGAAGAA
GGGTTAGGGAGTTGAGTGTAGGTTGGGGGAAGCACCTACAGGCTGCGAGAGGGATCA
AGGACGAACTCGTGAATGAGTACACTCCAGATCGCCAAGGATGTCTAGAAAAGACGAT
GTTGAATGGTGGAACCTTATCAGTGCCTAAGGATTACAGGACTGACGATTATTCTCTTG
TTCGTCTTGGCTGTGCCAAGACTAAAGGGAAGCATCGTGTAGTTACTATGCAATCGGCA
TTTGTGAAAAGGACTCTTACACCAGTACATCGCGCTCTTTATAATCACCTTAGCGATTTT
GACTGGCTCGTTCGTGGGGACGTCACCACTGGGGATTTCGAAGCTATTCTTCGAGATG
CAGAAAAGTATAAAGAACCGATTATTAGTGGCGATTACGATAGTGCCACTGATAATATCT
ATCAAAATGCGGTTCAATGCATCGTCGAGGAACTTTCGAAGGACAGTGACCTGACGGA
GGAGGAAAGGACAGTTTTTATGGGTTCTTTCGTAAACTTAAGGTACATAGATACCAAAA
CAATGGAAGTATTTCCAATTAAGCGAGGCTCAATGATGGGGAACTTATGTTCTTTCCCT
GTATTGTGTCTCCTTAACAAGGCATGTTTTGATATTGCAACTGACGAAGTTTACGGAAG
TGGTAACAAAAGAAAAGGAAGATTCAACGGTGATGATTGTATCTTCGCGGGCAGTACT
CGTATGTACCAATGTTGGAGAGAAGTTACTTCTAGGTATGGTCTTGTCGTAAATGAGTC
AAAGACAGACGTATCTCGTCGTTTCATCGATCTAAATAGTCAATGCTACGATATTGTTCG
CCGAAGAATGATCGGCAAACCAGTCTTGTCTTTTCTTGGACCCATAAACGATTCTGTAG
GAGAGCAGTTAACCTCTATCTTAGAGGGTATCTCTAGTCTGAAATGGGACGTCCAGCAA
TGGCTCGTCAATGTTCATGCTAGATACCTCGTCTCTTTGAAGGGTGTTAGCCTCTCTGG

AATCCCACAGGTCTGGGTGAAGCAGCTTGTAAAAAGGAAATGGTTCCGGAATGCGGTT
GTCTTAGGAGGTGCTGCTAAGGTCGTAAGACCTTACGTTCAGTACTCCTGGGTAAAGA
AAGGAGGTAAGAAAAGGGTCCGTGAGTGCACGGCAGTATTAAGAACCGAGACTAGAG
AACTCGATATGGTTATGGGACCTGTCCCAAAACCCGAATATCTCTCTAGAATCGACGAA
TTATGTTCTTCTATTCAGAACCTTCACACCGATTTTTGGTGTGGAAAGAATGTAATTGCT
GCAGTACCTACCCTTGACCGAAAAACCTTCAGACAAGAATACGACCGCAGACGGAAA
GCAGATTTCCCCCCCACGAGGAGGTGGTTTCTAGGATTTAGAGACCGGTTCGTGTACA
GATACCCCTCTGTGCTTTATCGAGAAATCGAGGATATGGATATTTTCTGGTCCCAAACTG
GGAGAGAAACTACCTATCCAGGATATTCGTCGAATATCAAGCTACAGCGGTGTATTTTG
TATCGCCCAAACCGATATGAAATCCGACCACCAACTCTACACTCGAATTCGCCACGCTA
TTCGCCCTTCTCTCAAGGTCTAAACCAGGTTTCCTTTTGGGATTGCCCAGTGAAGCGAG
AGAGACACGACGTGATTGACAAAGGCGCGTGATTCAAAGTGTAGATGGGAGAGACAG
AAAAACAAGAAAATAACAAAAAATGAGAGTGTCCTCTTGATCCGTATAATGATGGTAA
AGTTGACGAACCAACATTACGGCTGTTAAGAGATGGAGCAGGGAGATCTGTCCAATCC
GAGTAGCGATGACTCGGGGCATATGTGGGAGATGATAATACTCTTTCTTTTTTCACGTTT
TTATCACTAAACGACCGTTCCACGGTTACGAATCCCTATCTCGTATCGGGGTTGGACCAT
GAGTTTCCGCATTTTCTAATGTATAGCAATATACAAAAGATAATGTTGCCCTCATGTAAG
CCTAACCAATACGGTTATGGAGCACGGTTGTGCTTAATAGAGTTTCAATAGCGTGGTGG
CCCTGCCTGTTATCATGAGAACAGGTCCAGTTTGCGACTGGAAAAGTGGAGGCTATAC
GTGTTTAAGAATATTGAAAAGAAATTGAAAAAATTATCGACAATGAGAAACCAATATGT
CTCGTTTAATCGAACAAAAGAAGGGAACGATCGTCATGAAAAACCTGCACATCTAGAT
GGACATGATATACAGACAACCATAACAGTACTATAGATTGACAGAGTTCGCACGGAGTG
CGGAAAAATCGGCCTCTCATTTGATGTGACTTTTCTTGGCCTCGCC

>Contig 12923 (333 nt)
AGTACATAACTTATTTAGGAGGGTATGCTCCATTTAAAAAGATGTGTTCTTCAACATTTT
ATTATGCGACCAAAATATTGCCTGTGTCAGCCGTGTCAGTGAAAGGCGAAGCTTTGCC
ATAAAATAAATTGATAAATTACAAGTTGAGAACAGTGGTGGGATCACCAATAAACCAGT
ACATTCTGTCAACAATATGGAATTACGGGCCCAATCACATATTTGCATGGGAATCAACTC
CCATCAAGATTGGCATGCCCCTCAATGGTTACTGGATGACAAGTGTTTGGGCAATACAT
CCCAGGTGCCAATTCCATGTCAAGGGTGATTTTTCTG

>Contig 25026 (297 nt)
CCTTTATGAGAGACCCTGACCGCCCCTGGTCAAAGTATTCGCGCATGAAGTACATAACT
TATTTAGGTGGTTATGCTCCATTTAAAAAGCTGTGGGCTTCAACATTTAATTATGCGACC
CAAATATTGCCTGTGTCAGCCTTGTCCGTGAAAGGCGAAGCTTTGCCAGAAAAGAAAA
GGCTCAATGACAAGGTGAGAACAGAGGTGGGATCACCAATAACCCAGAACATTCTGTC
AACAAAATGGAATTACGGGCACAAAAACAGATATGCATGGGAATCAACTCCCATCAAG
ATTG

>Contig 46148 (344 nt)
TGAGCATGCAGCCCCACAAAAACGCGCTGCTCAAGCGCGCCTGGATACTAGTGCCATC
TGCTGTGACTTTCACATGCACTTGAGAAGCATGCAAACGGCTATCCGGGATGCGACGG
CAAACGTCAAGGGCTGCCCTGACAGCGACTGCCTCGGCAGCTTGCCTCGACATGCAG

GGAGCTAGCCACCAGCCAATGCACTGTGCCCCCCAGCTCAGCCATAGCAGCAGTCGCC
GGCTGGTTTAGTGCAATAGACGTTCTGCAATTGCCAGATCTGCAACTTGCAGTTGATCT
GATCACTCACTTTCTTCTCGTGACAATTTGGGCTTCTGTCCCACCCAATTTCCG
