# Supplementary material for: Four Novel Mycoviruses from the Hypovirulent Botrytis cinerea SZ-2-3y Isolate from Paris polyphylla: Molecular Characterisation and Mitoviral Sequence Transboundary Entry into Plants
Source: Viruses. 2022 Jan 14;14(1):151. doi: 10.3390/v14010151 (PMC8777694; doi:10.3390/v14010151)
Supplement: Supplementary file 1 [file viruses-14-00151-s001.zip › viruses-1505710-SI/Supplemantary File S2.pdf]

Supplementary File S2. The sequences of partial genome and encoded RdRp aa from BcFV8.

>Partial genome sequence of BcFV8 (GenBank acc. No. OL321741, 3074 nt)
TCATTGATTGTTGCTTGCCTAACATTTGTTTTTGTTTGGTCAGGCTTAGGAGTTGCTTTGTG
GGCCATGTTGGCGCTTTTAGTTTCTGGCAACTCTTTGTTCTTGTTGCCTGTTGTTGTGCTCG
GGCCTTGTGCCATCTTTGTCATCACATATGTTGTGTCTGGGACGATTGTAAATGAGGCAAA
TAATGTCAGGTTGCATGGCAAGAAGCGTGCCTTGTGGATTAGCACGGAGTTCAGTCCGCG
GAACCCAAGTGTCCAATTGGTAAGGTATGCGATTTGGAAGGGACATGATCAGACAGGAGA
ATGGCTCACCGCTGCTTTCAAGGTCTTTGACTTGCCTCCTGAACTGCAGGATGCCATTGGT
GACGGTATTGCGTCGCTTGGCTCTTATGCCTGGAGCGAGATGATACTTGGCATGGAGAAG
GTTGTGGACGGGCCTCAGTCCTTGTTCATTTCTCTTTCCGCATTTCTGTTTCTAGTTGCTTG
GGTGCCACAGACAGTCTGGAAGACCCTCAAGAAGTTTTACATCTTCCAGTTTGTCGTTTTC
ATCTGCCTTTGGCATTTGAAGATTGAGCATGTCCAGGCAATGGTTTTTGGCTTCATCCGGT
TTACTGTGTTTGCCTCCAAACATTTTAACCAAGACGACATTAGCCTTTTTTCTGAATGGGTT
AATTGGCGCCTCACTGCTGCATTTACCTCTGTTGCAGCATGGGCCACAGCACTCAATTCAG
AGGTGGCAAGATACCATTCAAAAAGCATTGGTGGTGGCTCTTCAAAATTGATCAGCCATT
TCAAGAATTTCACCATGCAGGGTGCCTTGTTCATTAGTGATTTGGGTTTGCCTTCCTTTGTG
CGGAGAAGGTTCAACCCTGAGGTGACAGTGAATGCTTTGCAGCAATCACTTGACATTATG
AAAGACCTTGGTTGGCCCATCAATGTGAAGATTGATGAGCCAGTTCCTATTGAAGGTTTTT
CACTGGAGTCATTCAAGGAATGGGTTTTGTGTGGTTCCGACCTCAAGACTGGTATTCACAA
TTGCAAGACTTATGTTGACAAAGACTTGTTGCACATTAAATCTGCTTTGGTCTACCGCCGG
ACAGAGGAGTATGCTAGCGAACTTAATGAAGTCACCGCTACAGCACGCTACTTCAAAAGG
CCTGATTATGAGTTCCCTGAGCTCCAATTGTCAGATGTGCGGCTTGTTATTGGGGACATTT
TCAGGCAATCCAAATTGACACCTTTCAATTACATCATCTCAAAGTGGGAGAAGAAGTATG
CTTTGGGTGCCTTTATGAGAGACCCTGMCCGCCCCTGGTCAAAGTATTCGCGCAAGAAGT
ACATAACTTATTTAGGTGGGTATGCTCCATTTAAAAAGCTGTGGGCTTCAACATTTTATTA
TGCGACCCAAATATTGCCTGTGTCAGCCGTGTCCGTGAAAGGCGAAGCTTTGCCAGAAAA
GAAATGGCTCAATGACAAGGTGAGAACAGTGGTGGGATCACCAATAACCCAGTACATTCT
GTCAACAATATGGAATTACGGGCCCAATCACAGATTTGCATGGGAATCAACTCCCATCAA
GATTGGCATGCCCCTCAATGGTTACTGGATGACAAGTGTTTGGGCAAGACATGCCAGGTG
CCAAGTCCATGTCCAGGGTGATTTTTCTGAATTCGATTCAACAGTGTCAGGAAAAGTCATT
GACATGATAAAAGCCGTAAGGAAGAAGGGTTTTGAGTTTCATCGAGACCGTGAAAGGATT
GCAGAATTAATAGATGCAAATTACTTTCAAGTCACAAATCAGCTTCTCAATACAACTTCAA
CTGGCAATGTCTATAAAAAGGGCACTGGCTTGACCACAGGCCATTCAAGTACTAGCATGG
ACAATTCCCTTGCTTGTGTGATTTTGTACCTGATGGCCTGGAAGGATATAACCGGGCTTTC
AGCCAAAGAATTCGTTCATTTCAATGAGCTTTCATGCTTTGGTGATGACCATGTCTTGTCA
TTCTTGGCAACCAAACCAGCCATTTGGACTCCCACAAACATATACAAAACTATGTTGAGGT
GGGGGCTTACAAACAATTTGGAAGTCATGAAGCTTGAGGACATTGAATTCCTTTCAAAGA
AGGGAAGGAAAGCTTCTGTCCCTGAAGTTTCGTGGTTGTCCTCTTTGGGCCTTAAGAATGT
TAGGTTTCTGGTATGGCATAACAAGTCAAAGTTGCTCGGGAAGCTGACTGCCAAGGTTAA
GAACATACAGCCTTCATACCGCGTCATTAGGCTGCTGAGCTACTTGTCTTTGACGGCGCAT
CATCAAGATGTGTATGATGGGATTGTGCATGCGATCACTTCCTCCAGGGCCATGATGGCA
ATGGTGAAAGCGAACGGCCTTGTCATTCCGTCTTACGAAAAGGTTGTGCGGGATTGGTATT

CTCCATCAGCAGCCCCCAAAGTCCCTGGTGATCTCATTGATGAAGATGAAAATTTTATCAA
TGGGCAGAGGATTGTCGAATATGGTAGCACAAACGCCCTTGACTCACTTGTTGGTGCTTTG
TCCATGTTGCCTGATTTGTTGTCCCCTGTTTTGTTCAATTACGGGTATGCAAAAGCTTTACA
GGTTTTCTTATTACCCAGGTTGTCCTGGGTTGTTGACTTCATGGCGGCTGCGAACAAAACA
AGTGCAGTTGGCGCTCTTCAGTACCAACTGTCCAAAACTCCTTACCGGTGGGTTGAAGTGC
CTTTGTTCGTGCCTGGTTCATCTTTTGCAAACAAGTCAAGCATGTTAGCAAGGCATTGGCT
TTACATGCTTTACATGTCGAACCGTCCAACCATGAAAACAGGTGCGTATCTCAACATGATA
ATCAACAGGATTGGCATACTCCAGTTTGTCATGAACGGGCGACTCTTCAACGAGCAAAAA
GATTCCGTCTTTTCACTGGACCAAGTCATAGTGTGTGCCATTTTGTCGTTTGTTGACATACC
TGACTGGTTTAGTGCAATAGACGTTCTGCAATTGCCAGATCTGCAACTTGCAGTTGATCTG
ATCACTCACTTTCTTCTCGTGACAATTTGGGCTTC
>Partial RdRp aa sequence of BcFV8（1020 aa）
ACLTFVFVWSGLGVALWAMLALLVSGNSLFLLPVVVLGPCAIFVITYVVSGTIVNEANNVRLHGKKRAL
WISTEFSPRNPSVQLVRYAIWKGHDQTGEWLTAAFKVFDLPPELQDAIGDGIASLGSYAWSEMILGMEKV
VDGPQSLFISLSAFLFLVAWVPQTVWKTLKKFYIFQFVVFICLWHLKIEHVQAMVFGFIRFTVFASKHFNQ
DDISLFSEWVNWRLTAAFTSVAAWATALNSEVARYHSKSIGGGSSKLISHFKNFTMQGALFISDLGLPSFV
RRRFNPEVTVNALQQSLDIMKDLGWPINVKIDEPVPIEGFSLESFKEWVLCGSDLKTGIHNCKTYVDKDLL
HIKSALVYRRTEEYASELNEVTATARYFKRPDYEFPELQLSDVRLVIGDIFRQSKLTPFNYIISKWEKKYAL
GAFMRDPXRPWSKYSRKKYITYLGGYAPFKKLWASTFYYATQILPVSAVSVKGEALPEKKWLNDKVRT
VVGSPITQYILSTIWNYGPNHRFAWESTPIKIGMPLNGYWMTSVWARHARCQVHVQGDFSEFDSTVSGK
VIDMIKAVRKKGFEFHRDRERIAELIDANYFQVTNQLLNTTSTGNVYKKGTGLTTGHSSTSMDNSLACVI
LYLMAWKDITGLSAKEFVHFNELSCFGDDHVLSFLATKPAIWTPTNIYKTMLRWGLTNNLEVMKLEDIEF
LSKKGRKASVPEVSWLSSLGLKNVRFLVWHNKSKLLGKLTAKVKNIQPSYRVIRLLSYLSLTAHHQDVY
DGIVHAITSSRAMMAMVKANGLVIPSYEKVVRDWYSPSAAPKVPGDLIDEDENFINGQRIVEYGSTNALD
SLVGALSMLPDLLSPVLFNYGYAKALQVFLLPRLSWVVDFMAAANKTSAVGALQYQLSKTPYRWVEVP
LFVPGSSFANKSSMLARHWLYMLYMSNRPTMKTGAYLNMIINRIGILQFVMNGRLFNEQKDSVFSLDQVI
VCAILSFVDIPDWFSAIDVLQLPDLQLAVDLITHFLLVTIWA
