# Supplementary material for: Four Novel Mycoviruses from the Hypovirulent Botrytis cinerea SZ-2-3y Isolate from Paris polyphylla: Molecular Characterisation and Mitoviral Sequence Transboundary Entry into Plants
Source: Viruses. 2022 Jan 14;14(1):151. doi: 10.3390/v14010151 (PMC8777694; doi:10.3390/v14010151)
Supplement: Supplementary file 1 [file viruses-14-00151-s001.zip › viruses-1505710-SI/Supplemantary File S3.pdf]

Supplementary File S3. The sequences of full-length genome and encoded complete RdRp aa from BcMV10, BcBoV18 and BcBoV19, respectively.

> Full-length genome sequence of Botrytis cinerea mitovirus 10 (GenBank OK634394, 2945 nt)
GGGGTCCTGACCATTTCTGATCAATAAATCCCGAGCTTTCGCTCTTTATTTATTTTAAGG
ATGATGTCCAGGACGTGATCTTGGTATGATCACCCAAGCGTATATACCGTTAGAACGAA
CCACCTGACTGTGTAGGGGAATTAACTGAATAACTACCCTGTTTTATAACAGTGGATAA
GATGAAATACATTCTACTTACAGTACGAACTTTCAGCTGGCAAGACTAATCATGAAGGC
TTGGCCCCTTACCACCGGATCTTGATTAAACTCACTTCTTTAGAAGGAAGTCTGTCAAG
ACGGCAATGAGAGACCTATACCTATAGCCCATCAAGTGATGGAATACTAAATCTTTAGA
CTTCTATGAAAACATCTACTTTAACAATTTTAAATTCTTTTAAATTATTAAAAAGACGTTC
TTTAAGAAGTTTCAAGATGGTATCTCTCGCCAAAATTGGCACGATCAAAAGCGTGTTCC
CAAAGTTAATTTCATTATCTTTGGGCCGCTTAGATCGTATTAAAGCAAGAGTCCGGATGT
CTAACAACTTGATACAGTTCATCCAGCGTATGTCTGTTCACCACGGTGACGCTTATACC
ATAAAATGGTTAAAGTGTTCTTCCGTAGCTTTACAGAAATACATTGCTGGTGATCCGATT
TCAAGTTTAAGATCTCTTGAACCTAAAATTCCTCTTCCACGGTTAATAAACGGTTGTCC
TAGTATAATTTCTAAGACAGATCGTAAGTTAATACGTGAAGGGAATACATGGGTGAGAC
GTTTTTGATTGACACAATTCGCTCTTTATAGGGTTTTAAAATTCCCTAGTAGAGTTGACT
TGTCATCGATCACAGATCCTTACTCAGGTTCAAGTTCCTTTCTGATACAGCAAATTCAG
TTGGCTAAGGATTTTAATCCTTTTGCAATATATGCAAAGGATTTCAAATTCCTTCCTCCA
ACTACCCTTGTTTTCTCTCAGAAGTCTTCTCCCTCCAATGGGATTTCCTATCAAGGAATT
CTTACGGATTATCATAACCTCTCTCGAGGTTCATGAATTTTAGGAGAGGACCTCGGGCC
TCGACTTGAGTGGCTAAATATAGTCGCTTATGTCGAAGTTTTGAGAAAATCAGGTTTGA
ATATGCGACGATGAGATTCTATCATCCATTCTCTTGATTCTCTTTTAGAGAATCTTAAGAT
TAATGGTATGAAATTACCTTCAAAGTCAAGTATCTTTGGAACTGGTTTATCTCAGTTCGC
GTTGAAACACGAGAGTGCTGGAAAGATTAGAATCTTCGCATTGCTGGATTCTATATCTC
AATCAGTACTTCGTCCTCTTCATGATTCCTTATTTGATATACTTAGATGTATTCCAAATGA
CGGAACTTTTGATCAGGACGCCTCGGTTTCCCGGTCTGCGGATAAACTGGCTAAGTATG
GAGTGGCATATTCTTTGGATCTTTCTTCTGCAACAGATAGGTTACCTTCAAGGCTAACA
GCCCAGATCCTACAGTCGATTTTATCGATTGAGGGATTTGGTGATGCTTGGCTTAAGGT
AATGATCGATAGAGACTTTTGTTTATCGTCTCTTGATCAAGTCTATGCATTAGAAGGTGA
AAAGTATATACCCCAATATTTTAGATATTCTGTGGGTCAGCCCATGGGTGGTCTAAGTTC
GTGAGCCGGATTAGCTATTACTCACCATTGGATCATGCAAGCGGTCTCTCTTCGGTGCT
CAAGAAAGTTTTCTAATCTTTCTTTGGTCACTTGAGAGGATCGTTACGAGGTCCTTGGT
GATGATATAGTTATCTTTGATGAAAATCTTGCTCAAGAGTACCTAATATTTATGAGAGAA
CTTGGTGTCGGTATAAACCTAACTAAGTCTCTTTCTAAATCATCAGATACTTTTGAGTTT
GCTAAAAGAACGATTAGTAGAGGTGTTAACATCTCTGGTTTATCGTTCCAACAAGCGCT
TAGTTCTTCATCTTTAGGATCACGAGTTTCAGATGCTTATACTTACAGCTCTTTAGGGCT
GGTAAGGACAGCATCTCATCTTGGACATCTATTGGCGGCAAAACCGACTTCATCTTCTT
TTAGGAAGATGAAAGAGATCGGTTTGCCAGCTCTGTCACTTTACAATCTTTTATTCTCA
AAAGAGATAATAGAGTTGGAAAGAGTACTAGAATGTATTGTCAATCCTCGATTTGAGGA
TTTCGACTTTGAGAAGGCCAAATTTGACCTTCCTTTGCATTCTATGCTACGACATTGCTT
AGATCTAATACGTCTAAAAGGGTCTAACACAAGTGAGTGAGGATTGGCTCCTTCCGGA
GTTCTTCCTTCTTCCTTGGCGTACCCCTTTTCTCGTCAGGATGACCGAGAAGAAGTCTC

TTCTGAGTACGAACCGCATTTAGTTGCAGTTATACTTCAGGAGGCTCTAGCTAAGTCTA
AAACCTTAGTTAGAGATTACGAGAACTTGATCAAGAGAGGTGCAGTTACTGTCTATAA
AGGACAGGGAACTAAACTTCTACAAGCTCAAGTCTCGGGTTTCTTCGAGGATCTAATT
ATTGAGTTTTCTGATTTAGATGTATCGGACTTCTGTGATGAAATAGAATCAATGCTATATC
GTCACGCGAAGTATCCTAAATACTCTATATCAGAAGCTCTCTCTACCTTAGATCGGGTAG
AGAATCTGATATTCCGTTTCACTTTTAAGACAGAGATGTCTCGTTCAAAATATGAACAA
GATTCTTCTCCGATCATAAAGATGTTGCGGAAATCAGAGGGTAGCATTCCCATTCCATAC
TGACAGACATCCGCTCTTCCTTAAATGATGATATATCATCCTTCTTAAAGGCGTTGACGA
ATTTCGTTAACGTCTCTAATTTAAAAGAGGGTCCCCAAGATTACTGTAAAAAGTATGCA
GGGGGAGTGCCCTTCATATTTCATGAGAATGCT

>RdRp aa sequence of Botrytis cinerea mitovirus 10 (818aa)

MKTSTLTILNSFKLLKRRSLRSFKMVSLAKIGTIKSVFPKLISLSLGRLDRIKARVRMSNNLI
QFIQRMSVHHGDAYTIKWLKCSSVALQKYIAGDPISSLRSLEPKIPLPRLINGCPSIISKTDR
KLIREGNTWVRRFWLTQFALYRVLKFPSRVDLSSITDPYSGSSSFLIQQIQLAKDFNPFAIYA
KDFKFLPPTTLVFSQKSSPSNGISYQGILTDYHNLSRGSWILGEDLGPRLEWLNIVAYVEVL
RKSGLNMRRWDSIIHSLDSLLENLKINGMKLPSKSSIFGTGLSQFALKHESAGKIRIFALLDS
ISQSVLRPLHDSLFDILRCIPNDGTFDQDASVSRSADKLAKYGVAYSLDLSSATDRLPSRLT
AQILQSILSIEGFGDAWLKVMIDRDFCLSSLDQVYALEGEKYIPQYFRYSVGQPMGGLSSW
AGLAITHHWIMQAVSLRCSRKFSNLSLVTWEDRYEVLGDDIVIFDENLAQEYLIFMRELGV
GINLTKSLSKSSDTFEFAKRTISRGVNISGLSFQQALSSSSLGSRVSDAYTYSSLGLVRTASHL
GHLLAAKPTSSSFRKMKEIGLPALSLYNLLFSKEIIELERVLECIVNPRFEDFDFEKAKFDLP
LHSMLRHCLDLIRLKGSNTSEWGLAPSGVLPSSLAYPFSRQDDREEVSSEYEPHLVAVILQE
ALAKSKTLVRDYENLIKRGAVTVYKGQGTKLLQAQVSGFFEDLIIEFSDLDVSDFCDEIES
MLYRHAKYPKYSISEALSTLDRVENLIFRFTFKTEMSRSKYEQDSSPIIKMLRKSEGSIPIPY
WQTSALP

> Full-length genome sequence of BcBoV18 (GenBank OK634395, 2759 nt)

CAGGTTAGCACGCTCAACGTGCATTCCCGATACCGACGATAGGATGAAACCTTCAGAC
GATTCTATTGGGAATTCAGGGAATACCAGAAATGGAGGTACCTGTACCCGCTGCCCAGC
AGCTGGGTCCCTGTCAATTGCTCTCACACGAGCAGTTCGTATCATACGATTGGAATATA
AGATTCCGAATGATATTTTACTTCCTAGGAAGCAGAACTGCACGAGCCTAAGAGCTTCA
TGGAATGAGTGGACGACTTCAGCACAAAGCTGTGTGAATGTCAATAAGCCTAGAGGTA
GAAGGCTAGCTATGTTGGTTAAGTCCGGCAACCGGCTATTCGATCTGCCTTGCAGAAGC
TGTGACAAGTCACTAAGCTGGCAAGCAGAACAGGACTGGATAAGGAGCGTGGGTAAG
CCAACTCCTAACGATCTTGTACCAACGATCTTTGATCTCTTTAAACTCAGAGAGAAGGT
CAGACAAGAAGTCAGAGGATGGGGAAGGAGACTCGCATCGGCGAGAGACAGTTTGG
AGGAACCGTGTTTGGGCGACTACGTCCCGGGGGTTCAAGGGTGTGCAGAATTGAACA
CAATGAGTGGCGGAACCTTAGGAGTCTCAGAGAGTGAATATAGCGGCCAGCGCAATGT
GGTCAGACTGGGATGTGCAAAAACAAAGGGAAAGTTTCGTGTTGTGACAATGCAATC
TGCGGAGGTCAAGAGAGTCTTGACTCCACTGCATAATGCCTTGTATGGACACATCTCAT
CGAAAGGATGGTGTGTCCGGGGGGACGTTTTAAGGGGGGACTTCGAGGAGATTATCG
AAGATCGAAAGGAAGGTGAGAAGTATATTAGTGGCGACTATAAGAGTGCTACTAACAA
AATTTATACAGAAGCTGTACTAACGATAGTCGATGAGATCTCCAGGACCCCTGAGCTCA
CAGAATTGGAGCGAGAAGTGTTTATGGAATCCTTTACGGATATGTGGTGGTTCAAGAG

ACTTGAGAGCGGACCTATGTGTAGGGGCTCTCCCATGGGAAGTTTGGTAAACTTCCCG
ATGCTGTGTATCTTGAACAAAGCTTGTTTTGAAATCGCCTGCGATAACGTAATAGGCTT
GCGCCGCAAAGAAGTAAAGAGGGTGAAGATTAACGGAGACGACATAATGTTCGCCGG
CACCGCTGTTTTGTACCAGGAATGGCGACGTGTGACCGGAATTTATGGCCTTGAAGTTA
ATGAAAGTAAAACCGAGATCTCAGATAGGTGGTTGGACCTGAATAGTCAGTCCTTCGA
TACCTTTCGGAGAAAGATGGTCGCAAAGGCGACACTCGGATTTCTTCGCCCTAATCGA
CAAGAACCAGGCGCATTGTTGCGCGCCATCATTGTGGGTATGAAAGGTTTTAAGACCG
GTCATATCATGCAAGTGATCACTGTGCTCCGGCACGAAGTGGCTTTGCGTGGTGTAACT
GAGGATCTTACGGAAATCGGACCCTATTGGCGAAACGCTCTCGTGAAGAAGAAGTGGT
TTCGGCAAGCACTTGCAGTTGGCAAGTGCCCCGAAATCAGACGCGGTCAAGACCGGT
CGCTGGCAGTTGAGCCAGGGCCACCTCCACGTGAACGATTCTATGCAGTTATTACCCGC
ATCGCCGCGCAGGCGCAGAGTGATAACACAAAGGAGTGGACAGGCGTTAAGGTAAAG
AACCTTACTGTTCGCCTAGACAGAAAAGCATGGAAGAATTTGACAAAGGAAAAAGCT
GACCTCGCCGATAGGAGGTATATTTGGAAAGGAGTCCGTTGGAGTTTCTTGTGGCCCA
AGGAATTACTCACCCTAGTCAGGGAGTATTTTCCCCAGATACTGGAAACCAAGACGCC
AAAATGGATGACTGACCACCCTTTCTTAACGCGCTCACCTGTAGGTCGCGTTGAGAAG
AAAAGAAAATATGCCTCATGCTCTCCCCCCTCGTGTCTCCAACCTGTGGAGTTTGTTAC
GAAGTGGGAAAGGGAACAGGCTGAATGGGCAAAGGAAATGTCGATGTCTGTGTAGAC
GGTGGTCTCACGAGGACGTCATGAAATTATGGGCCGAATGCTTGATTCGAACCGATTCA
AGAGGTGTCCACGAAAGGAGTCGCATAAGGATGGTAAGTCGGATTGCCAGTACGGCTC
ACCGCAGCAGAGTAGGGAGACTCACTATATCATTATATATTGGCCTCAAAGAACTGAAA
TGACAGACGAAACTGGGAGAGTTTTAACGAAGGGAAGTGCTCTAATGGTTAACGGTA
GGATCGCTAGTGGGTATCCAGCTATTTAAGTGGATAGAAATCCGAAAGGACGTTAAAA
ATTGTCAACCGGTGACCCTCGTGAGAGGTGAGCTAACGGGGCGGATAAGTCCTCGTCG
ATCATTGTAGACCGCATTAGAAGTACCAGCGTAGTTAGATCTCGTCAATGGAAAACCAA
GCGCACGGGGTTTCAGGAAATGAGTGTAAAGTCAATCGCTTGATAGGGGTCGTTATGG
AAAACCTATATGCTGATTAACGGTGTTGTACTAAAGACTGGTAACGTATGCCTGCATAC
GAGTGCCGCACGGAGTGCGATGACGATTGGAGTGGTTCCTTAAGATGACCGTAAGGAT
GACGGTAGAGAGGAGTTAATGGATTGACTAGTGAGTCACCGATGCAATTTCATCGGATT
TCACTGCAGATGTTGCAGACCAGCGGCAATGGATCCTGGAGTCAACGCGAAAGCGTG
GGTGCCCGCTACCCCCGGGGTAGACACCA

> RdRp aa sequence of Botrytis cinerea botoulivirus 18 (661aa)

MKPSDDSIGNSGNTRNGGTCTRCPAAGSLSIALTRAVRIIRLEYKIPNDILLPRKQNCTSLRA
SWNEWTTSAQSCVNVNKPRGRRLAMLVKSGNRLFDLPCRSCDKSLSWQAEQDWIRSVG
KPTPNDLVPTIFDLFKLREKVRQEVRGWGRRLASARDSLEEPCLGDYVPGVQGCAELNT
MSGGTLGVSESEYSGQRNVVRLGCAKTKGKFRVVTMQSAEVKRVLTPLHNALYGHISSK
GWCVRGDVLRGDFEEIIEDRKEGEKYISGDYKSATNKIYTEAVLTIVDEISRTPELTELEREV
FMESFTDMWWFKRLESGPMCRGSPMGSLVNFPMLCILNKACFEIACDNVIGLRRKEVKR
VKINGDDIMFAGTAVLYQEWRRVTGIYGLEVNESKTEISDRWLDLNSQSFDTFRRKMVAK
ATLGFLRPNRQEPGALLRAIIVGMKGFKTGHIMQVITVLRHEVALRGVTEDLTEIGPYWRN
ALVKKKWFRQALAVGKCPEIRRGQDRSLAVEPGPPPRERFYAVITRIAAQAQSDNTKEWT
GVKVKNLTVRLDRKAWKNLTKEKADLADRRYIWKGVRWSFLWPKELLTLVREYFPQILE
TKTPKWMTDHPFLTRSPVGRVEKKRKYASCSPPSCLQPVEFVTKWEREQAEWAKEMSMS
V

> Full-length genome sequence of Botrytis cinerea botoulivirus 19 (GenBank OK634396, 2812 nt)
AGATTCAAGCCTTTTGAGCTTGTTTCCGCATTCCGATCGTGTCGAAGTTGCATTATGAG
TAACCATTCGTCATTGATCAATGCCGCGCCTACCTGTCAGGGTGGAGCGTCTCTTGTCA
AGTCTCTTAAAGTTGTAGCTGAAACTGTGTCTCGTGAATTTGGATTCACCACAGGCCCT
TGTATAAAGGGTGTCAACTGCATAACCATAAGAGAAGAATGGGATGGATGGATCAAGG
ATTGTCTTGCCAACCAAGTTTATTGGAGCCGAGTGCGACATGCACCCAGCAGAAAAAG
AAAGTTTGTTTCCCTGTCAAAGGGGGTCAAGCGAGTATTTGATGCTACTTGCAAACAG
TGCGACAAAAAAGCCTCGAGAGAGGCAATCAATAAATGGACAAAGAAAATGGCTGAG
GATTGCCGTGATGGGGAAACCCATTGCTCCAGGTACCTTGATCAGTTGAGAAGAAGGG
TTAGGGAGTTGAGTGTAGGTTGGGGGAAGCACCTACAGGCTGCGAGAGGGATCAAGG
ACGAACTCGTGAATGAGTACACTCCAGATCGCCAAGGATGTCTAGAAAAGACGATGTT
GAATGGTGGAACCTTATCAGTGCCTAAGGATTACAGGACTGACGATTATTCTCTTGTTC
GTCTTGGCTGTGCCAAAACTAAAGGGAAGCATCGTGTAGTTACTATGCAATCGGCATTT
GTGAAAAGGACTCTTACACCAGTACATCGCGCTCTTTATAATCACCTTAGCGATTTTGA
CTGGCTCGTTCGTGGGGACGTCACCACTGGGGATTTCGAAGCTATTCTTCGAGATGCA
GAAAAGTATAAAGAACCGATTATTAGTGGCGATTACGATAGTGCCACTGATAATATCTAT
CAAAATGCGGTTCAATGCATCGTCGAGGAACTTTCGAAGGACAGTGACCTGACGGAG
GAGGAAAGGACAGTTTTTATGGGTTCTTTCGTAAACTTAAGGTACATAGATACCAAAAC
AATGGAAGTATTTCCAATTAAGCGAGGCTCAATGATGGGGAACTTATGTTCTTTCCCTG
TATTGTGTCTCCTTAACAAGGCATGTTTTGATATTGCAACTGACGAAGTTTACGGAAGT
GGTAACAAAAGAAAAGGAAGATTCAACGGTGATGATTGTATCTTCGCGGGCAGTACTC
GTATGTACCAATGTTGGAGAGAAGTTACTTCCAGGTATGGTCTTGTCGTAAATGAGTCA
AAGACAGACGTATCTCGTCGTTTCATCGATCTAAATAGTCAATGCTACGATATTGTTCGC
CGAAGAATGATCGGCAAACCAGTCTTATCTTTTCTTGGACCCATAAACGATTCTGTAGG
AGAGCAGTTAACCTCTATCTTAGAGGGTATCTCTAGTCTGAAATGGGACGTCCAGCAAT
GGCTCGTCAATGTTCATGCTAGATACCTCGTCTCTTTGAAGGGTGTTAGCCTCTCTGGA
ATCCCACAGGTCTGGGTGAAGCAGCTTGTAAAAAGGAAATGGTTCCGGAATGCGGTTG
TCTTAGGAGGTGCTGCTAAGGTCGTAAGACCTTACGTTCAGTACTCCTGGGTAAAAAA
AGAAGGTAAGAAAAGGGTCCGTGAGTGCACGGCAGTATTAAGAACCGAGACTAGAGA
ACTCGATATGGTTATGGGACCTGTCCCAAAACCCGAATATCTCTCTAGAATCGATGAATT
ATGTTCTTCTATTCAGAACCTTCACACCGATTTTTGGTGTGGAAAGAATGTAATTGCTG
CAGTACCTACCCTTGACCGAAAAACCTTCAGACAAGAATACGACCGCAGACGGAAAG
CAGATTTCCCCCCCACGAGGAGGTGGTTTCTAGGATTTAGAGACCGGTTCGTGTACAG
ATACCCCTCTGTGCTTTATCGAGAAATCGAGGATATGGATATTTTCTGGTCCCAAACTGG
GAAAGAAACTACCTATCCAGGATATTCGTCGAATATCAAGCTACAGCGGTGTATTTTGT
ATCGCCCAAACCGATATGAAATCCGACCACCAACTCTACACTCGAATTCGCCACGCTAT
TCGCCCTTCTCTCAAGGTCTAAACCAGGTTTCCTTTTGGGATTGCCCAGTGAAGCGAG
AGAGACACGACGTGATTGACAAAGGCGCGTGATTCAAAGTGTAGATGGGAGAGACAG
AAAAACAAGAAAATAACAAAAAATGAGAGTGTCCTCTTGATCCGTATAATGATGGTAA
AGTTGACGAACCAACATTACGGCTGTTAAGAGATGGAGCAGGGAGATCTGTCCAATCC
GAGTAGCGATGACTCGGGGCATATGTGGGAGATGATAATACTCTTTCTTTTTTCACGTTT
TTATCACTAAACGACCGTTCCACGGTTACGAATCCCTATCTCGTATCGGGGTTGGACCAT
GAGTTTCCGCATTTTCTAATGTATAGCAATATACAAAAGATAATGTTGCCCTCATGTAAG

CCTAACCAATACGGTTATGGAGCACGGTTGTGCTTAATAGAGTTTCAATAGCGTGGTGG
CCCTGCCTGTTATCATGAGAACAGGTCCAGTTTGCGACTGGAAAAGTGGAGGCTATAC
GTGTTTAAGAATATTGAAAAGAAATTGGAAAAATTATCGACAATGAGAAACCAATATGT
CTCGTTTAATCGAACAAAAGAAGGGAACGATCGTCATGAAAAACCTGCACATCTAGAT
GGACATGCTATCCAGACAACCCTAACAGTACTATAGAATGACAGAGTTCGCACGGAGT
GCGGAAAAATCGGCCTCTCATTTGATGTGACTTTTCTTGGCCTCGCCAATGAAAAACC
AT

> RdRp aa sequence of Botrytis cinerea botoulivirus 19 (694 aa)

MSNHSSLINAAPTCQGGASLVKSLKVVAETVSREFGFTTGPCIKGVNCITIREEWDGWIKD
CLANQVYWSRVRHAPSRKRKFVSLSKGVKRVFDATCKQCDKKASREAINKWTKKMAED
CRDGETHCSRYLDQLRRRVRELSVGWGKHLQAARGIKDELVNEYTPDRQGCLEKTMLN
GGTLSVPKDYRTDDYSLVRLGCAKTKGKHRVVTMQSAFVKRTLTPVHRALYNHLSDFDW
LVRGDVTTGDFEAILRDAEKYKEPIISGDYDSATDNIYQNAVQCIVEELSKDSDLTEEERTV
FMGSFVNLRYIDTKTMEVFPIKRGSMMGNLCSFPVLCLLNKACFDIATDEVYGSGNKRKG
RFNGDDCIFAGSTRMYQCWREVTSRYGLVVNESKTDVSRRFIDLNSQCYDIVRRRMIGKP
VLSFLGPINDSVGEQLTSILEGISSLKWDVQQWLVNVHARYLVSLKGVSLSGIPQVWVKQL
VKRKWFRNAVVLGGAAKVVRPYVQYSWVKKEGKKRVRECTAVLRTETRELDMVMGPV
PKPEYLSRIDELCSSIQNLHTDFWCGKNVIAAVPTLDRKTFRQEYDRRRKADFPPTRRWFL
GFRDRFVYRYPSVLYREIEDMDIFWSQTGKETTYPGYSSNIKLQRCILYRPNRYEIRPPTLH
SNSPRYSPFSQGLNQVSFWDCPVKRERHDVIDKGA
