# Supplementary material for: Four Novel Mycoviruses from the Hypovirulent Botrytis cinerea SZ-2-3y Isolate from Paris polyphylla: Molecular Characterisation and Mitoviral Sequence Transboundary Entry into Plants
Source: Viruses. 2022 Jan 14;14(1):151. doi: 10.3390/v14010151 (PMC8777694; doi:10.3390/v14010151)
Supplement: Supplementary file 1 [file viruses-14-00151-s001.zip › viruses-1505710-SI/Supplementary Figures and Tables.pdf]

Table S1. List of the oligonucleotide primers used in this study.

Function	Primer name	Sequence (5'-3')	Target PCR product
Detect Primer for mycoviruses	Contig111-F	CATACTTAGCCAGTTTATCCG	587
	Contig111-R	CAATTCGCTCTTTATAGGGTT	
	Contig334-F	CTTATCCGCCCCGTTAGCTC	578
	Contig334-R	GGCCCAAGGAATTACTCACC	
	Contig420-F	GTCCAACCCCGATACGAGA	523
	Contig420-R	CCCCTCTGTGCTTTATCGAGA	
	Contig25026-F	CCTTTATGAGAGACCCTGACC	260
	Contig25026-R	TTTTGTGCCCGTAATTCCA	
Synthesize genome middle fragment cDNA of mycoviruses	111M-F	TATGAAAACATCTACTTTAACA	2452
	111M-R	AGAGCGGATGTCTGTCAGTAT	
	334M1-F	TTCTATTGGGAATTCAGGGA	1899
	334M1-R	GGTTGGAGACACGAGGGGGGAG	
	334M2-F	CAGGCGCAGAGTGATAACACA	953
	334M2-R	ACGGTCATCTTAAGGAACCAC	
	420M1-F	CCCTTGTATAAAGGGTGCCAA	1731
	420M1-R	TCCTCGATTTCTCGATAAAGCA	
	420M2-F	CTCTCTGGAATCCCACAGGTC	1342
	420M2-R	GCGAGGCCAAGAAAAGTCACA	
Universal primer for terminal sequences of mycoviruses	RACE-OLIGO	(PO4)CATTGCATCATGATCGATCGAATT CTTTAGTGAGGGTTAATTGCC-(NH2)	
	O5RACE-1	GGCAATTAACCCTCACTAAAG	
	O5RACE-2	TCACTAAAGAATTCGATCGATC	
	O5RACE-3	CGATCGATCATGATGCAATGC	
BcMV10 genome terminal specific primers	111-R1	ATTAACTTTGGGAACACGCTTT	486 (O5RACE-2)
	111-R2	TTGAAACTTCTTAAAGAACGTC	452 (O5RACE-3)
	111-F1	AATCAATGCTATATCGTCACGC	290 (O5RACE-2)
	111-F2	ATCGGGTAGAGAATCTGATATT	301 (O5RACE-3)
BcBoV18 genome terminal specific primers	334-R1	CCATCCTCTGACTTCTTGTCT	430 (O5RACE-2)
	3R-122	AGGAAGTAAAATATCATTCGG	202 (O5RACE-3)
	3F-1768	TACTGGAAACCAAGACGCCAA	300 (O5RACE-2)
	3F-2474	AATCGCTTGATAGGGGTCGTT	287 (O5RACE-3)
BcBoV19 genome terminal specific primers	420-R1	AGGTTCCACCATTCAACATCG	450 (O5RACE-2)
	4R-215	CTCAGCCATTTTCTTTGTCCA	387 (O5RACE-3)
	4F-1613	AGACAAGAATACGACCGCAGA	818 (O5RACE-2)
	4F-2623	TGAGAAACCAATATGTCTCGT	192 (O5RACE-3)
The primers for BcFV8 partial genome sequence	BcFV8-1549-F	TCATTGATTGTTGCTTGCCTA	1549
	BcFV8-1549-R	TTTTGTGCCCGTAATTCCA	
	BcFV8-1786-F	CCTTTATGAGAGACCCTGACC	1876
	BcFV8-1786-R	GAAGCCCAAATTGTCACGAGA	
Identification for *Botrytis cinerea*	ITS1-F	TCCGTAGGTGAACCTGCGG	539
	ITS4-R	TCCTCCGCTTATTGATATGC	
	Bc-F	CAGGAAACACTTTTGGGGATA	327
	Bc-R	GAGGGACAAGAAAATCGACTAA	

Table S2. Sequence identities of BcFV8 to other viruses used for multiple sequence alignment and phylogenetic analysis.

Name	Abbreviation	aa identity (%)	GenBank Acc. No.	Classification (Genus / Family)
Botrytis cinerea fusarivirus 6	BcFV6	87.2 (889/1020)	QJT73721.1	
Botryosphaeria dothidea fusarivirus 1	BdFV1	53.7 (543/1012)	QHI00151.1	
Botrytis cinerea fusarivirus 5	BcFV5	84.8 (865/1020)	QJT73719.1	
Erysiphe necator associated fusarivirus 3	EnaFV3	85.2 (869/1020)	QKN22708.1	*Fusarivirus /Fusariviridae*
Macrophomina phaseolina single-stranded RNA virus 1	MpsRV1	51.2 (520/1016)	ALD89094.1	
Monilinia barnavirus J	MbVJ	85.6 (873/1020)	QED43004.1	
Rutstroemia firma fusarivirus 1	RfFV1	55.1 (561/1018)	AZT88659.1	

Table S3. Sequence identities of BcMV10 to other viruses used for multiple sequence alignment and phylogenetic analysis.

Name	Abbreviation	aa identity (%)	GenBank Acc. No.	Classification (Genus / Family)
Fusarium boothii mitovirus 1	FbMV1	54 (426/788)	BBG56023.1	*Fungal Mitovirus / Mitoviridae*
Nigrospora oryzae mitovirus 1	NoMV1	50 (395/783)	AZP53928.1	
Plasmopara viticola lesion associated mitovirus 49	PvlaMV49	46 (360/788)	QIR30272.1	
Soybean leaf-associated mitovirus 5	SlaMV5	35 (252/716)	ALM62240.1	
Botrytis cinerea mitovirus 3	BcMV3	37 (222/593)	YP_009182161.1	
Alternaria alternata mitovirus 1	AaMV1	32 (252/782)	QDB74990.1	
Botrytis cinerea mitovirus 1	BcMV1	21 (134/642)	YP_002284334.2	
Botrytis cinerea mitovirus 6	BcMV6	28 (166/597)	QHJ68502.1	
Botrytis cinerea mitovirus 2	BcMV2	18 (121/671)	YP_009182160.1	
Botrytis cinerea mitovirus 4	BcMV4	19 (134/711)	YP_009182163.1	
Botrytis cinerea mitovirus 5	BcMV5	19 (138/711)	QJT73704.1	
Sclerotinia sclerotiorum mitovirus 4	SsMV4	23 (106/465)	QJQ28895.1	
Nigrospora oryzae mitovirus 2	NoMV2	17 (108/621)	AZP53929.1	
Fusarium circinatum mitovirus 2-1	FcMV2-1	16 (105/647)	AHI43534.1	
Solanum chacoense mitovirus 1	ScMV1	19 (115/591)	DAB41743.1	*Plant Mitovirus / Mitoviridae*
Beta vulgaris mitovirus 1	BvMV1	16 (97/591)	AVH76945.1	
Chenopodium quinoa mitovirus 1	CqMV1	15 (107/730)	YP_009551903.1	
Oxybasis rubra mitovirus 1	OrMV1	17 (101/604)	DAB41745.1	
Erigeron breviscapus mitovirus 1	EbMV1	19 (111/590)	DAB41748.1	
Cannabis sativa mitovirus 1	CsMV1	15 (91/600)	QQO58813.1	
Ocimum basilicum RNA virus 2	ObRV2	19 (124/648)	YP_009408146.1	
Solanum tuberosum	*S. tuberosum*	24 (74/310)	XP_006364252.1	*Plant mitochondrion*
Eucalyptus grandis	*E. grandis*	25 (63/246)	XP_039157828.1	
Capsicum annuum	*C. annuum*	18 (54/307)	XP_016567141.1	
Anthurium amnicola	*A. amnicola*	37 (94/254)	JAT57754.1	
Pseudo-nitzschia australis	PnA	26 (105/397)	CAE0710133.1	
Solanum chacoense	*S. chacoense*	31 (72/230)	JAP35587.1	

Table S4. Sequence identities of BcBoV18 to other viruses used for multiple sequence alignment and phylogenetic analysis.

Name	Abbreviation	aa identity (%)	GenBank Acc. No.	Classification (Genus / Family)
Botrytis cinerea botoulivirus 19	BcBoV19	40 (177/448)		*Botoulivirus / Botourmiaviridae*
Botrytis cinerea ourmia-like virus 17	BcOlV17	78 (518/661)	QJT73683.1	
Sclerotinia sclerotiorum ourmia-like virus 3	SsOlV3	70 (448/642)	AWY11006.1	
Botrytis cinerea ourmia-like virus 16	BcOlV16	71 (460/648)	QJT73682.1	
Botrytis cinerea ourmia-like virus 12	BcOlV12	34 (213/631)	QJT73678.1	
Sclerotinia minor botoulivirus 1	SmBV1	36 (196/541)	QHR78948.1	
Botrytis ourmia-like virus	BOlV	35 (192/555)	YP_009182165.1	
Epicoccum nigrum ourmia-like virus 1	EnOlV1	40 (257/641)	QDB75003.1	
Botrytis cinerea ourmia-like virus 8	BcOlV8	21 (96/452)	QJT73674.1	*Magoulivirus / Botourmiaviridae*
Botrytis cinerea ourmia-like virus 7	BcOlV7	18 (81/450)	QJT73673.1	
Penicillium citrinum ourmia-like virus 1	PcOlV1	17 (75/430)	AYP71797.1	
Magnaporthe oryzae ourmia-like virus	MoOlV	19 (104/536)	SBQ28480.1	
Rhizoctonia solani ourmia-like virus 1	RsOlV1	19 (92/480)	ALD89131.1	
Pyricularia oryzae ourmia-like virus 3	PoOlV3	15 (79/538)	BBF90578.1	*Scleroulivirus / Botourmiaviridae*
Sclerotinia sclerotiorum ourmia-like virus 1	SsOlV1	17 (80/467)	ALD89138.1	
Soybean leaf-associated ourmiavirus 2	SlaOV2	18 (77/420)	YP_009666498.1	
Botrytis cinerea ourmia-like virus 1	BcOlV1	16 (95/592)	QJT73667.1	*Penoulivirus / Botourmiaviridae*
Phoma matteucciicola ourmia-like virus 1	PmOlV1	18 (101/560)	QIP68359.1	
Pyricularia oryzae ourmia-like virus 1	PoOlV1	20 (107/529)	BBF90576.1	
Cassava virus C	CVC	15 (61/399)	YP_003104770.1	*Ourmiavirus / Botourmiaviridae*
Epirus cherry virus	EcV	14 (89/654)	YP_002019754.1	
Ourmia melon virus	OmV	15 (62/419)	YP_002019757.1	
Rhizoctonia solani ourmia-like virus 3	RsOlV3	10 (53/529)	QDW65428.1	*Rhizoulivirus / Botourmiaviridae*
Rhizoctonia solani ourmia-like virus 2	RsOlV2	12 (71/598)	QDW65427.1	
Armillaria mellea ourmia-like virus 1	AmOlV1	12 (59/498)	QUD20355.1	

Table S5. Sequence identities of BcBoV19 to other viruses used for multiple sequence alignment and phylogenetic analysis.

Name	Abbreviation	aa identity (%)	GenBank Acc. No.	Classification (Genus / Family)
Botrytis cinerea botoulivirus 18	BcBoV18	40 (177/448)		*Botoulivirus / Botourmiaviridae*
Botrytis cinerea ourmia-like virus 17	BcOlV17	38 (195/511)	QJT73683.1	
Sclerotinia sclerotiorum ourmia-like virus 3	SsOlV3	32 (204/636)	AWY11006.1	
Botrytis cinerea ourmia-like virus 16	BcOlV16	36 (192/528)	QJT73682.1	
Botrytis cinerea ourmia-like virus 12	BcOlV12	78 (537/687)	QJT73678.1	
Sclerotinia minor botoulivirus 1	SmBV1	77 (528/683)	QHR78948.1	
Botrytis ourmia-like virus	BOlV	29 (174/600)	YP_009182165.1	
Botrytis cinerea ourmia-like virus 8	BcOlV8	18 (100/558)	QJT73674.1	*Magoulivirus / Botourmiaviridae*
Botrytis cinerea ourmia-like virus 7	BcOlV7	16 (77/481)	QJT73673.1	
Penicillium citrinum ourmia-like virus 1	PcOlV1	16 (94/579)	AYP71797.1	
Magnaporthe oryzae ourmia-like virus	MoOlV	20 (95/485)	SBQ28480.1	
Rhizoctonia solani ourmia-like virus 1	RsOlV1	15 (92/606)	ALD89131.1	
Pyricularia oryzae ourmia-like virus 3	PoOlV3	15 (73/481)	BBF90578.1	*Scleroulivirus / Botourmiaviridae*
Sclerotinia sclerotiorum ourmia-like virus 1	SsOlV1	16 (59/376)	ALD89138.1	
Soybean leaf-associated ourmiavirus 2	SlaOV2	30 (70/232)	YP_009666498.1	
Botrytis cinerea ourmia-like virus 1	BcOlV1	11 (58/523)	QJT73667.1	*Penoulivirus / Botourmiaviridae*
Phoma matteucciicola ourmia-like virus 1	PmOlV1	21 (79/376)	QIP68359.1	
Pyricularia oryzae ourmia-like virus 1	PoOlV1	26 (82/321)	BBF90576.1	
Cassava virus C	CVC	13 (87/646)	YP_003104770.1	*Ourmiavirus / Botourmiaviridae*
Epirus cherry virus	EcV	15 (85/569)	YP_002019754.1	
Ourmia melon virus	OmV	14 (76/546)	YP_002019757.1	
Rhizoctonia solani ourmia-like virus 3	RsOlV3	12 (67/549)	QDW65428.1	*Rhizoulivirus / Botouliviridae*
Rhizoctonia solani ourmia-like virus 2	RsOlV2	10 (57/603)	QDW65427.1	
Armillaria mellea ourmia-like virus 1	AmOlV1	13 (77/571)	QUD20355.1	

Table S6. Primers used for construction of pXT1/BcMV10 clone and detection of BcMV10 sequences in cucumber plants

Function	Virus	Primer name	Primer sequence (5-3)	PCR product (bp)
cDNA clone construction for BcMV10	BcMV10	F	TTTCATTTGGAGAGGGGGGTCCTGACCATTTCTG (1-19bp)	~3kb
		R	ATGCCATGCCGACCCAGCATTCTCATGAAATATG (2927-2945bp)	
Detection of positive clone of BcMV10	TXR/BcMV10	TXR-CX	TCAACAAAGGGTAATATCGGGAA (pXT1 vector primer)	~0.8kb
		R	ATGAAATTAACTTTGGGAACACG	
	BcMV10/NOS	F	ATCCTAAATACTCTATATCAGAAGC	~0.5kb
		NOS-R	ACCCATCTCATAAATAACGTCATGC (pXT1 vector primer)	
	pXT1/BcMV10	TXR-CX	TCAACAAAGGGTAATATCGGGAA (pXT1 vector primer)	~3.5kb
		NOS-R	ACCCATCTCATAAATAACGTCATGC (pXT1 vector primer)	
Detection of BcMV10 in Cucumber plants	BcMV10-1	F	CATACTTAGCCAGTTTATCCG	~0.6kb
		R	CAATTCGCTCTTTATAGGGTT	
	BcMV10-2	F	GTACCCCTTTTCTCGTCAGG	~0.4kb
		R	TACCCTCTGATTTCCGCAAC	
	BcMV10-3	F	TAATACGTGAAGGGAATACATGG	~0.7kb
		R	ATAAAATCGACTGTAGGATCTGG	
	BcMV10-4	F	TAATACGTGAAGGGAATACATGG	~1.7kb
		R	GTTCTCGTAATCTCTAACTAAGG	
		R	ATAAAATCGACTGTAGGATCTGG	

Note: The underlined sequence is reverse complementary to the pXT1 vector sequence.

Table S7. Predicated NLSs in mycoviruses RdRp using the online website (http://nls-mapper.iab.keio.ac.jp)

Mycovirus	Position	Sequence	Type
BcMoV18	619	VGRVEKKRKYA	Monopartite NLS
	621	RVEKKRKYASCSPPSCLQPVEFVTKWERE	Bipartite NLS
BcMoV19	76	PSRKRKFVSL	Monopartite NLS

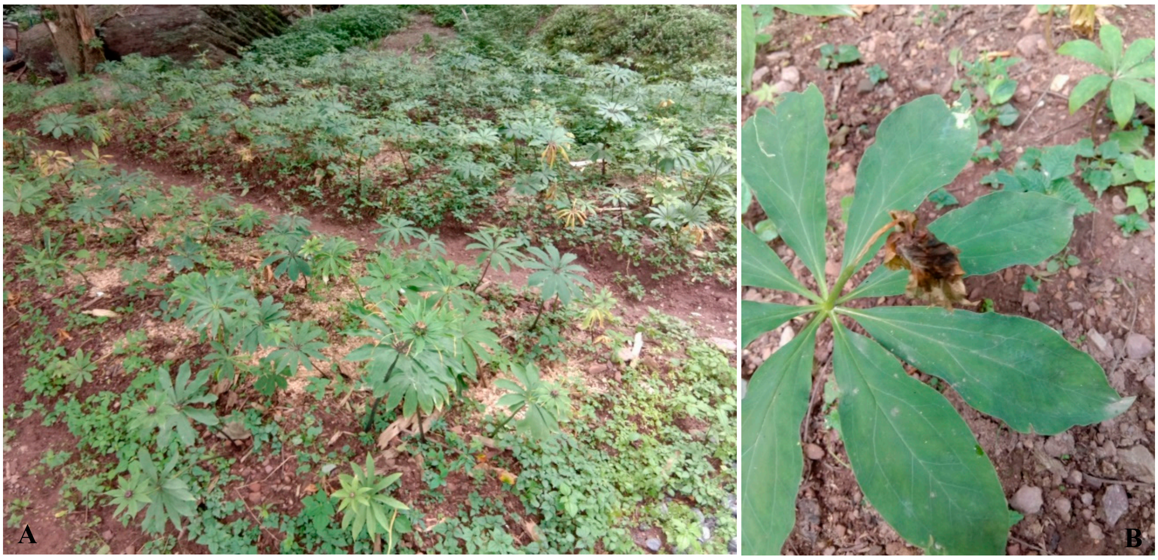

Supplementary Figure S1. The symptoms of *Paris polyphylla* plants infected with grey mold diseases as a whole (A) and the grey mold symptoms on single *P. polyphylla* plant (B).

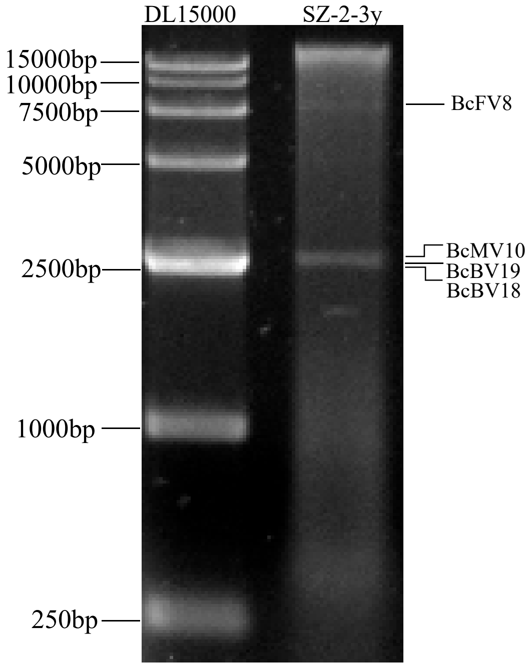

Supplementary Figure S2. 1.2% agarose electrophoreses analysis of dsRNAs

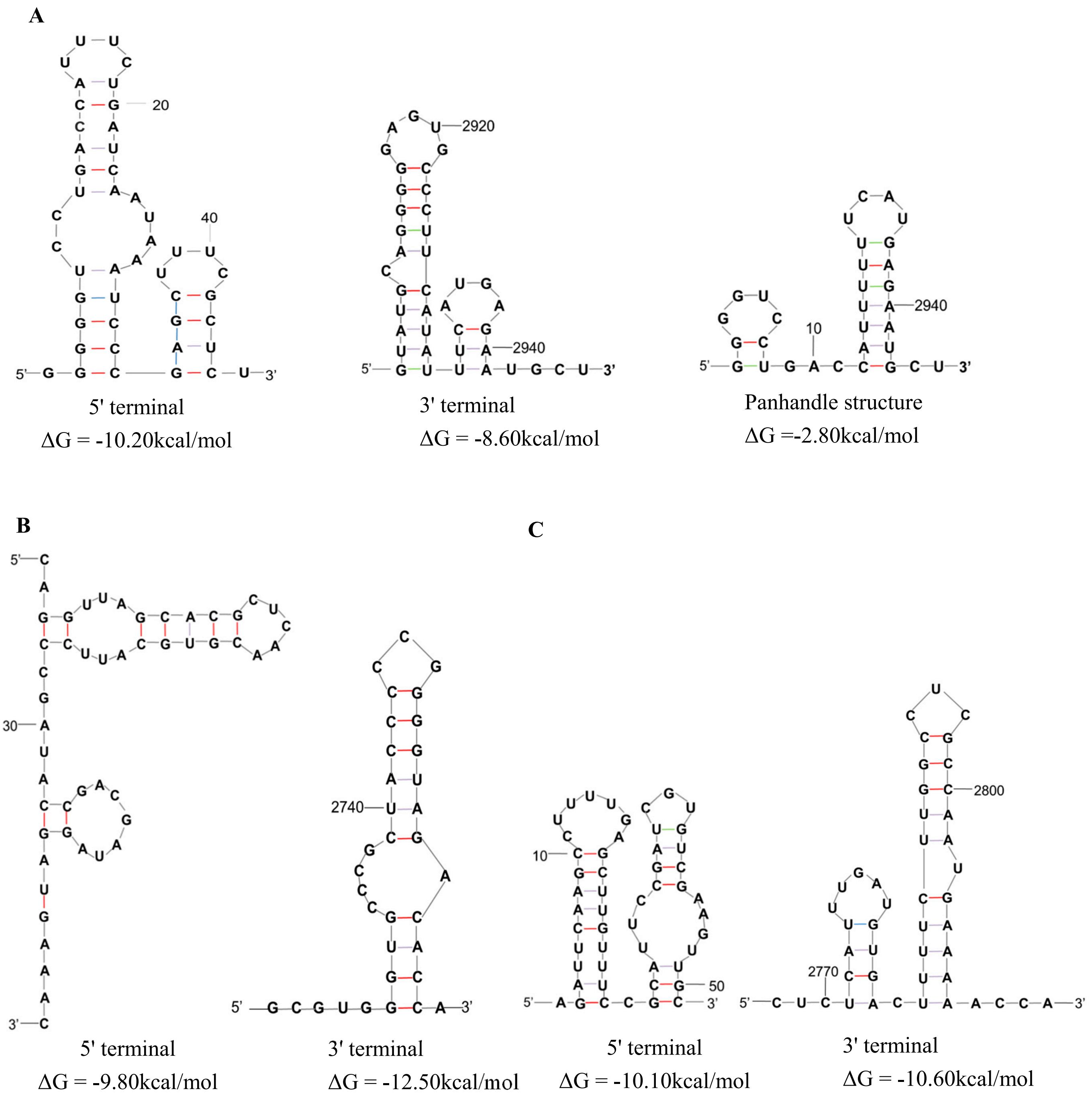

Supplementary Figure S3. The stem-loop second structures of terminal, non-coding RNA regions of BcMV10 (A), BcBoV18 (B) and BcBoV19 (C), respectively.

A

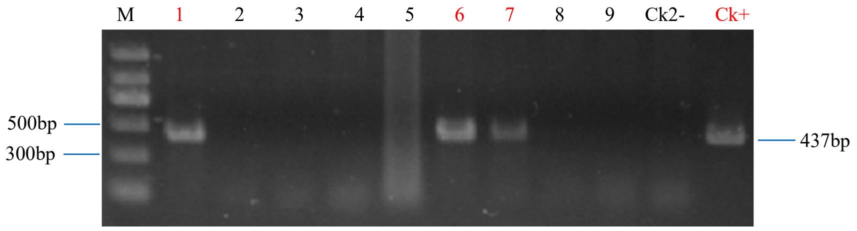

B

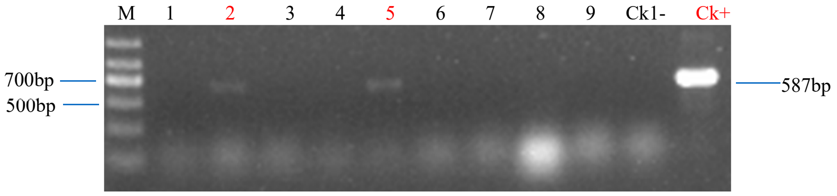

Supplementary Figure S4. The BcMV10 sequences from the new leaves of 'Cuiyu' cv inoculated with SZ-2-3y mycelia (A), and agro-infiltration with pXT1/BcMV10 clone (B) were detected by RT-PCR, respectively. Line 1–9: total RNAs of the leaves from 9 'Cuiyu' cv plants, respectively, were extracted as templates to detect BdMV10; Ck2-: total RNA from 'Cuiyu' cv plant inoculated by empty PDA as negative control; Ck1-: pXT1-inoculated plants as negative control; Ck+: Total RNA from SZ-2-3y mycelia on PDA; M: Marker III.

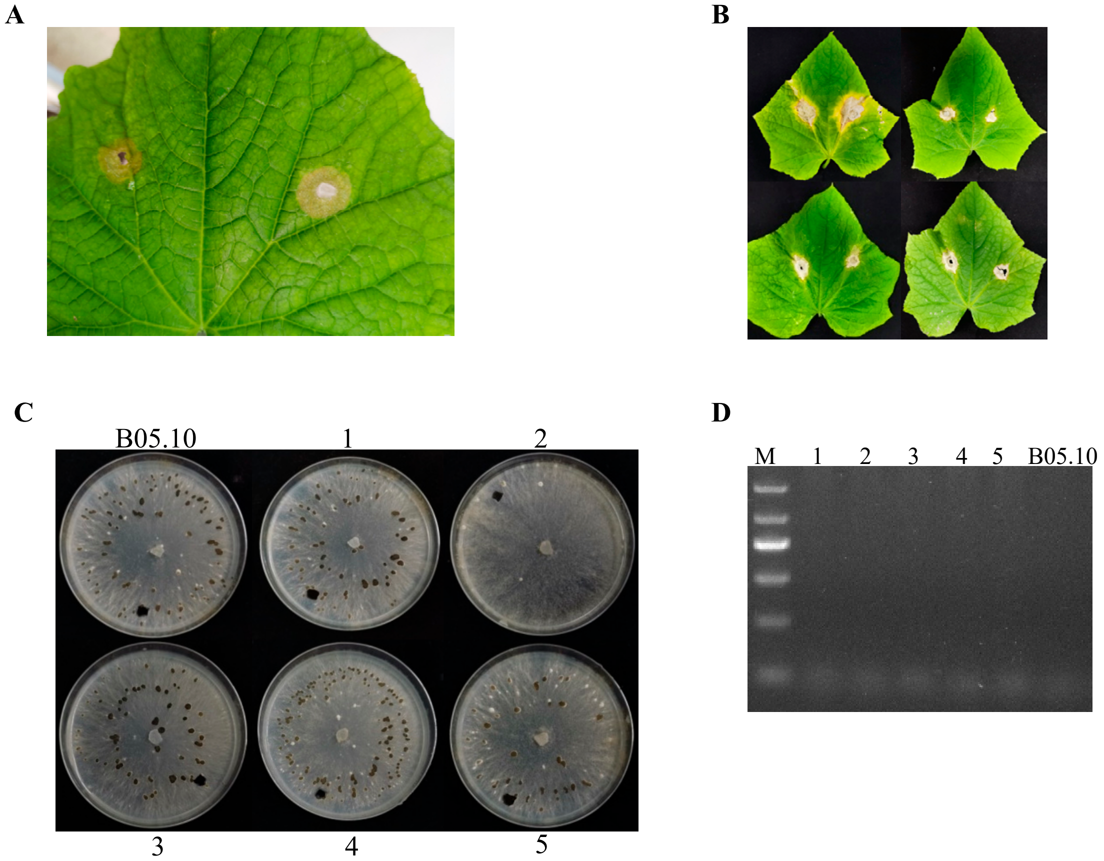

Supplementary Figure S5. Schematic diagram of intact inoculation of B05.10 strain on the new leaves of cucumber plants and RT-PCR detection of the BcMV10 sequence from the isolated strains. The symptoms were exhibited on new leaves of 'Cuiyu' cucumbers at 2 days (A) and 7 days (B) post-inoculation of B05.10 strain; the colony morphology of strains isolated from the leaves at two weeks post-inoculation of B05.10 strain (1–5, the isolated strains) (C); BcMV10 sequences from 5 isolated strains were detected by RT-PCR (D).
